# Clinical Notes on Characteristics

**Published:** 1888-02

**Authors:** C. Carleton Smith, Edmund J. Lee

**Affiliations:** Philadelphia; Philadelphia


### CLINICAL NOTES ON CHARACTERISTICS.

C. Carleton Smith, M. D., } Philadelphia.

Edmund J. Lee, M. D., }

### ACTEA SPICATA.

Mental: There is chiefly mental depression, melancholy and anxiety. All symptoms are worse from fright and anxiety. Its chief pains are of a rheumatic nature. In the face there is a pulling, *tearing* pain extending from decayed tooth to temples; worse from slightest touch or movement of the muscles.

In limbs, right wrist pains, intolerably, is swollen; motion is impossible; slightest pressure on palm of hand near little finger causes patient to cry out. Its rheumatic pains are chiefly in the small joints, which are very tender to the slightest touch.

Swelling of *joints after slight fatigue.* (Farrington puts it thus: "The patient goes out feeling tolerably comfortable, but as he walks the joints ache and even swell.") The pains are chiefly of a *tearing* kind. Actea-sp. follows well after Nux vomica. In rheumatic complaints compare with Bry., Cimic., Puls., etc.

(NOTE: Caul., Natr-p., Sal-ac., and Sticta are prominent remedies for rheumatic troubles in the small joints.)

### ÆSCULUS HIPPOCASTANUM.

MENTAL: Generally depressed, low-spirited; is irritable, loses temper easily, regains control slowly. (Loses temper easily, but regains it quickly, Sulph.) Confusion of senses; the headache is generally frontal, of a dull kind or a pressure. Dull frontal pain, followed by flying stitches at epigastrum (headache alternating with pain in abdomen, Cina, Plb.); with sensation of constriction of skin on forehead. Headache with drowsiness, feels too weak to walk.

(NOTE: Sense of constriction or contraction of skin on forehead, Æsc-h. Amm-c., Apis, *Arn.*, Bapt., Caul., Hura, Mill., Phys., Sep., Verat. Drowsiness with headache in general, Acon., Agar., Ars., Bruc., Camph., Clem., Con., Creos., Crotal., Cyc., Gels., Hipp., Hydr., Ign., Indig., Lach., Lobel., Mur-ac., Nat-s., Nitr., Nux-m., Phos., Ran-b., Stann., Stront., Strych., Sul-ac.; with pains in forehead, Ailan., Asar., Chin-s., Gins., Indig., Indium, Laur., Nat-s., Nux-j., Op., Stann., Tanac., Zinc.)

FACE looks pale and miserable. Face swells after washing it; rubbing it after washing produces red spots under the skin. (Heat with red spots after washing, Phos.)

TEETH feel as if covered with oil. Toothache better from warm drinks and pressing teeth together; worse from cold drinks.

(NOTE: Warm drinks relieve, Lyc., Nux-m., Nux-v., Rhus, Sulph.; pressing teeth relieves, Bell., Brom., Chin., Bry., Ign., Natr-m., Phos., Puls., Rhus; worse from cold drinks, Calc., Cham., Caust., Hepar, Lach., Merc., Nat-m., Nux-m., Nux-v., Puls., Sabad., Sil., Staph., Sulph.)

THROAT: Burning; dryness; feels dry and stiff when swallowing. Frequent inclination to swallow, with dry, constricted fauces. Dryness after eating. Tonsils dark, congested and swollen, worse left side (Apis); inclination to swallow with dull aching, burns like fire.

(NOTE: This burning in throat is peculiar with some drugs, but varies in degree. Thus, we find: burning as from alcohol, Amm-c.; as if inflamed, Nat-m.; like pepper, Mezer., Sal-ac., Verat.; like peppermint, Elaps.; like a red-hot ball, *Phyt.*)

STOMACH, ETC. : Pain, continuing *until he eats again.* Burning at epigastrium and feeling as if about to vomit, removed by eating dinner. Also distended feeling after eating ; regurgitation of food an hour after eating.

Violent burning in stomach, bowels, and liver, with distress, nausea, and violent vomiting. Stitches at the epigastrium, following the headache ; much dull aching in region of liver.

(NOTE : Dinner ameliorates, *Anac.* Chel., Cinnab. Eating ameliorates, Amm-m., *Anac.*, Bov., Cann-s., Canth., Chel., Ferr., Gamb., Graph., Ign., *Iod.*, Kali-c., Laur., Mezer., Nat-s., Nit-ac., Nux-v., Petr., Phos., Puls., Sabad., Stront. Dull aching in abdomen and stomach, increases as it becomes empty ; eating relieves, Fago. Feels better after a full meal, Ars., *Iod.*, Phos. Symptoms disappear during dinner, begin anew two hours after, *Anac.* Bœnninghausen gives amelioration while eating, Alum., Ambr., *Anac.*, Caps., Chel., Croc., *Ign.*, *Lach.*, Mezer., Spig., *Zinc.*)

ANUS, ETC : *Excessive dryness* of rectum ; *dry*, uncomfortable feeling in rectum, feels as if it were *filled with small sticks.* Mucous membrane feels thickened, which seems to obstruct feces. *Burning*, *fullness*, *pressure*, and *itching* at anus ; *soreness*, *burning*, and *itching* at anus. Large *purple* colored hemorrhoids with burning, looking like ground nuts and very painful ; apt to be accompanied with severe, *dull* backache. Hemorrhoids generally " blind." Pains worse when walking. "Severe pain in anus ; could not sit, stand, or lie, (only possible position was kneeling). The pain was like a knife, sawing back and forth ; almost a martyrdom for agony."—*Hughes.*

(NOTE : The dryness, burning, feeling as if filled with small sticks, the fullness, the large, purple hemorrhoids with the severe backache—these comprise a group of symptoms found nowhere else. This drug is *not* a remedy for " piles," but when indicated it will cure its peculiar kind of hemorrhoids.)

STOOL : Constipation ; stool hard, dry, passed with difficulty ; followed by severe pain and backache. Protrusion after stool. Stool first part hard, last softer and white as *milk.*

URINE hot ; burns as it passes. Frequent and scanty ; clear or more frequently dark brown.

LEUCORRHŒA, with lameness across sacro-iliac articulations, and great fatigue from walking, because that part of back gives out in walking even a little way. Dark, thick corroding leucorrhœa, with constant backache. This same backache and giving out is seen in pregnant females. Also, piles very troublesome during pregnancy.

Respiratory Organs: Sensation of dryness and stiffness of the glottis (Chlorum.) and of laryngeal muscles. Hacking cough caused by constriction of fauces. Chronic cough, with emaciation.

Hot sensation in chest; spits blood in morning on rising, (before rising Nit-ac., Nux-v.) Stitches in lower left lobe, *relieved on passing flatus.*

Back: *Constant dull aching*, affecting sacrum and hips, very much aggravated by walking, motion, and stooping, almost impossible to walk or to rise from sitting. This backache accompanies most all the complaints of this drug.

Legs, etc.: Back and legs weak, can hardly walk. Knees ache, muscles sore; worse on waking and motion. (Bry.)

Peculiarities: Sensation of fullness, as from too much blood, in ears, nose, heart, lungs, stomach, brain, skin, etc. Mucous membranes feel *dry, swollen*, or burn. Muscles sore or bruised, especially in moving or walking, and from motion. (Abrot., Bry.) The stitches in left lower lung *relieved by passing flatus* is very peculiar. Compare: Aloe, Collin., Merc., Nux-v., Podo., Sulph.

### ÆTHUSA.

Mental: Delirium; imagines she sees rats, dogs, etc.; wants to jump out of bed or the window. Bad humor; irritability; great anxiety and restlessness, followed by violent pains in head and abdomen. Weeping and crying; more so as disease progresses. After social conversation all the symptoms disappear. Wine aggravates.

Head: Vertigo, with sleeplessness; cannot raise the head. Looking upward increases the vertigo and the headache. As the giddiness ceases the head becomes hot.

Headache, pressure, pulsation, stitches, etc. Pain in the forehead, as if it would split; at its height vomiting, and finally diarrhœa; thin stools relieving the headache.

Pains are relieved by passing of flatus (also Cann-i., Cic.), temporarily by eating and by pressure. Constant sensation as if pulled by the hair. Unable to hold head up. (See Acet-ac.) Headaches appear periodically, after checked menses; are worse walking and looking upward.

Distressing pain in occiput, nape, and down spine, relieved by friction with hot whisky.

(Note: Diarrhœa with headache, Con., Glon., Graph., Nitr., Stram.; in forehead, Agar. Vomiting with headache, Cadm., Caust., Con., Dulc., Graph., Kali-c., Lach., Mosch., Natr-m.,

Nux-m., Rhus-r., Stram., Viper-t.; in forehead, Crotal., Iris, Phos., Verat-v.)

EYES: Pupils dilated, eyes staring, eyeballs turned down (in convulsions).

FACE: Features express great anguish and pain, pale, *well marked linea nasalis.*

Herpetic eruption on end of nose. (On nose, Ham., Nat-c., Nit-ac., Spig.)

THROAT AND MOUTH: *Aphthœ* in mouth and throat, making the patient very miserable. (Aphthæ, *Ars.*, *Borax*, *Lach.*, *Merc.*, *Sul-ac.*, etc. Intensely sore and raw mouth, Arum-t. Infant's sore mouth, better after beginning to nurse, Bry. Child cries when nursing, Borax.) Feeling as if tongue were too long. So long cannot use it—presses against teeth.—*Guernsey.* (Tongue feels too long, Acon., Mur-ac., Sumbul; feels too large, Par., Puls.)

VOMITING, ETC.: Children or pregnant women who vomit a frothy substance as white as milk.

Eructations of food an hour after eating it, in adults (after every meal, Nat-m., Phos.; during pregnancy, Acet-ac.). *Intolerance of milk.*

Sudden and violent vomiting of food (in children). The milk, curdled or not, is *forcibly* ejected as soon as taken, then follows weariness and drowsiness. (Drowsy after vomiting also, Ant-t., Ipec.)

Adults may vomit with a feeling of great distress, which they cannot describe, or may complain of a distress as if stomach were turned upside down, with burning up to chest.—*Guernsey.*

(NOTE: In vomiting of children Æthusa occupies a very characteristic position. The child vomits everything soon after taking it, but more particularly *milk.* The vomit and stool are apt to be alike. The vomit may be white, yellowish, or greenish curds. It is very forcibly ejected, and after it the child sleeps; it *awakes hungry,* and eats again only to vomit at once. With Ant-cr. the child vomits sour milk in little white lumps, *but refuses to nurse again,* and has the *white* tongue. With Ant-tart., vomiting is followed by drowsiness, languor, loathing, and desire for cool things; lying on right side relieves the vomiting. Calcarea has vomiting of *sour* curds as soon as milk is taken, or milky curds in the stool; large, fat babies with sour sweat; milk disagrees. Milk is cheesy, Cham. Milk tastes salty, child refuses it, Calc-p. Aversion to the milk, refuses to nurse, or, if it does so, vomits the milk, Sil. Milk is yellow and bitter, hence baby rejects the breast, Rheum. Baby refuses to take the breast, Calc-p., Cina, Merc., Sil., Stann. Child cries on attempting to take breast, Ant-t.)

STOMACH, ETC.: Painful contractions, so severe as to prevent vomiting. Great distress with the vomiting. Colic followed by vomiting, vertigo, and weakness. Sensation of coldness in abdomen.

STOOL, ETC.: Stools generally loose and green, bilious, or of curdled milk, like the vomit.

Obstinate constipation, with worrying and fretting. Pain and tenesmus before and after stool. Semi-comatose condition after stool, also after vomiting.

URINARY: Cutting pains in the bladder, with frequent desire to urinate. Nocturnal enuresis, with vomiting of curdled milk after nursing; greenish, watery diarrhœa.

FEMALE ORGANS: Menses checked by warm bath. Lancinating pains in sexual organs. Eruption of pimples on external parts, which itch as she gets warm.

RESPIRATORY: The sufferings of the patient render him almost speechless; the disease has a tendency to deprive him of speech entirely. Respiration often becomes hoarse and hissing; very difficult and painful respiration. Sensation as if chest were encircled by a band, which causes difficult breathing.—*Guernsey*.

HEART: Weak, intermittent pulse, with palpitation and headache.

NERVES, ETC.: Epileptiform spasms, with clenched thumbs (also Ars., Arum-t., Caust., Cocc., Glon., Hell., Hyos., Secale, Stann.), eyes turned down (also Canth., Cham., Ether), dilated, staring; immovable pupils, foam at mouth, teeth set, pulse small, hard, and quick.

(NOTE: Hyos. has "child sickens after eating; vomits, or shows signs of distress at stomach; sudden shriek, and then insensible." For the spasms of children Guernsey gives the following hints: Caused by fall or injury, Arn. Child lies as if dead, pale but warm, finally it twists its mouth from side to side, a violent jerk seems to pass through whole body, and then respiration and consciousness gradually return, Arsenic. Starts from sleep with wild look, dilated pupils, red, flushed face, sleepy after spasm, Bell. After suppressed measles, Bry. After suppressed catarrh of head or chest, Camphor. Child seems well, when suddenly it becomes rigid (Cina, Ipec.), then relaxation with much prostration, also violent jerks through body, Cicuta. Child is very excitable and weak, and in consequence frequently suffers with spasm, as from excessive playing and laughing, Coffea. Spasms, commencing in extremities and preceded by vomiting, after convulsion child screams, turns and twists in all directions, till another spasm occurs, Cuprum. After retrocession of the rash in scarlatina, Cupr.-acet. Body has bluish tint, and muscles of back, face, and jaws are chiefly affected, Hydro-acid. Spasms returning at same hour every day, single parts seem to be affected, screaming and trembling, Ignatia. Spasms caused by swelling of gum over a tooth, Creosote. Much gasping for breath either before, during, or after the spasm, there may also be a bluish tint on skin, Laur. Swelling of gums, salivation, etc., or after suppressed salivation, Merc. Screaming before and during spasm in infants, or from fright, Opium. Spasms which return at the change of the moon, Silicea. Child seems afraid, shrinks from objects, and spasm from failure of eruption to come out fully, Stram. Pale skin, difficult breathing (also Secale), and spasms from retrocession of eruptions, Ant-tart. When a cold sweat comes on forehead during or after the spasm, cough before or after spasm, sometimes syncope after, Veratrum. Pale children (especially during teething), cross, irritable for days, cries out in sleep. rolls head from side to side (Secale), right side twitches, abdomen distended, and more urine than usual, Zinc.)

SLEEP: Sleep of Æthusa is restless. Patient is drowsy after stool or vomiting. On falling asleep there is rolling of eyes or slight convulsions.

PECULIARITIES: Anxiety and painful distress; great weakness, especially in children (child complains of being tired all the time, Cina); emaciation; want of power to hold head up or to stand. *Intolerance of milk;* black tongue and bilious diarrhœa in typhus; lancinating pains; sleep disturbed by violent startings; after much vomiting and purging child becomes cold, clammy, semi-unconscious, and will lie with staring eyes and dilated pupils. Sweat relieves the malaise and tendency to delirium.

COMPARE Ant-c., Ars., Asar., Calc., Cic., Con., Cupr., Ipec., Op.

### AGARICUS EMETICUS.

Most marked is the vertigo, which is so severe one must be carried to bed; is not able to sit or stand.

Sudden, violent longing for ice-cold water (during the worst attacks of anxiety), *which causes gradual relief.* (Anxiety partially relieved by cold drinks, Acon., Sulph.)

Violent vomiting, sensation as if stomach hung on threads, which would be torn, with ice-cold sweat on face; faintness even from moving head or listening to reading. Vinegar unbearable. (Aggravation from vinegar, *Ant-c.*, Ars., Bell., Brom., Ferr., Sep., Sulph.,)

CONDITIONS : *Cold water relieved speedily and permanently.*

